# Antibacterial Activity of Oregano (*Origanum vulgare* L.) Essential Oil Vapors against Microbial Contaminants of Food-Contact Surfaces

**DOI:** 10.3390/antibiotics13040371

**Published:** 2024-04-18

**Authors:** Loris Pinto, Salvatore Cervellieri, Thomas Netti, Vincenzo Lippolis, Federico Baruzzi

**Affiliations:** Institute of Sciences of Food Production, National Research Council of Italy, Via G. Amendola 122/O, 70126 Bari, Italy; loris.pinto@ispa.cnr.it (L.P.); salvatore.cervellieri@ispa.cnr.it (S.C.); th.netti97@gmail.com (T.N.); vincenzo.lippolis@ispa.cnr.it (V.L.)

**Keywords:** oregano essential oil, natural antimicrobials, foodborne pathogens, HS-GC-MS analysis, cross-contamination, abiotic surfaces

## Abstract

The antimicrobial effect of eight essential oils’ vapors against pathogens and spoilage bacteria was assayed. *Oreganum vulgare* L. essential oil (OVO) showed a broad antibacterial effect, with Minimum Inhibitory Concentration (MIC) values ranging from 94 to 754 µg cm^−3^ air, depending on the bacterial species. Then, gaseous OVO was used for the treatment of stainless steel, polypropylene, and glass surfaces contaminated with four bacterial pathogens at 6–7 log cfu coupon^−1^. No viable cells were found after OVO treatment on all food-contact surfaces contaminated with all pathogens, with the exception of *Sta. aureus* DSM 799 on the glass surface. The antimicrobial activity of OVO after the addition of beef extract as a soiling agent reduced the *Sta. aureus* DSM 799 viable cell count by more than 5 log cfu coupon^−1^ on polypropylene and glass, while no viable cells were found in the case of stainless steel. HS-GC-MS analysis of the headspace of the boxes used for the antibacterial assay revealed 14 different volatile compounds with α-Pinene (62–63%), and p-Cymene (21%) as the main terpenes. In conclusion, gaseous OVO could be used for the microbial decontamination of food-contact surfaces, although its efficacy needs to be evaluated since it depends on several parameters such as target microorganisms, food-contact material, temperature, time of contact, and relative humidity.

## 1. Introduction

Essential oils (EOs) are natural extracts composed of different monoterpenes, monoterpenoids, sesquiterpenes, and other volatiles (esters, ketones, aromatic phenols, alcohols, aldehydes, ethers, hydrocarbons, coumarins, and organic acids) [1]. They are endowed with antifungal [2,3] and antibacterial activity [4]. A large number of existing studies focus on antibacterial activity based on direct contact with pathogenic and spoilage bacteria [5,6]. Recent studies showed the antibacterial activity of oregano and thyme essential oils in the vapor phase against *Listeria monocytogenes* on culture medium and radish sprouts [7,8]. In particular, the antibacterial action of the EO vapors was defined using a pH-differential medium [9], with different airtight experimental apparatus [7,8,9]. However, the development of vapor-phase plate assays is necessary in order to evaluate the antibacterial activity of a large number of EOs with common multi-well plates.

The application of EO vapors in active packaging systems controlled bacteria growth on foods of animal and vegetable origin. However, negative effects on food quality are sometimes associated with EO exposure [10]. It is well known that food-contact surfaces (e.g., stainless steel, glass, and plastic surfaces) can be contaminated by spoilage and pathogenic bacteria, becoming the source of food cross-contamination phenomena [11,12,13]. Several studies evaluated the decontamination efficacy of gaseous ozone [14], UV-C light, and atmospheric plasma jets [15] on different food-contact surfaces contaminated with bacterial pathogens. These methods have the drawbacks of the long-term exposure necessary for bacterial inactivation [14], the rise of surface temperature during the treatment [15], and the need for specific equipment for the decontamination.

Conversely, the application of chemical preservatives and disinfectants usually requires simpler equipment and user-friendly approaches. Within the huge chemicals available today, in recent years, the application of natural extracts and compounds has been of great interest in several fields.

For these reasons, the application of liquid EOs was proposed as a natural decontamination strategy for abiotic surfaces [16,17]. In particular, orange peel EO determined a 5 log cfu piece-1 reduction of *Salmonella typhimurium* and *Escherichia coli* viable cells on stainless steel surfaces [16], as well as *Thymbra capitata* L. EO significantly reduced *E. coli* load on different food-contact materials after 10 min of contact [18]. Essential oil compounds (EOCs) were also evaluated for their antibacterial activity on food-contact surfaces, as demonstrated by Thymol and Carvacrol against *Staphylococcus aureus* on stainless steel [19]. However, their application should be carefully evaluated because of possible inductive biofilm production effects, as in the case of oregano EO against *Sta. aureus* sessile cells [20]. However, their efficacy could be reduced on non-flat surfaces and junctions, which are difficult to treat. To the best of our knowledge, the antibacterial efficacy of EO vapors is not sufficiently reported. For this reason, the decontamination of food-contact surfaces by EO vapors is proposed in this work.

The application of EO vapors was considered to verify their possible antimicrobial action on the most important food-contact surfaces: glass, polypropylene, and stainless steel. These materials are commonly applied in a huge number of cases, such as industrial food equipment, countertops for tables, sinks, refrigerators, etc. The same food-contact surfaces, including those used for food packaging, are also present in home kitchens. Therefore, the reduction of microbial viability resulting from natural compounds released from EOs could be useful to counteract the cross-contamination phenomena in both industrial and domestic environments.

In this study, a multi-well plate assay has been developed to assess the antibacterial activity of EOs in the vapor phase against spoilage and pathogenic bacteria. Then, stainless steel, polypropylene, and glass food-contact surfaces, contaminated with four pathogenic bacteria, were treated with the most active EO in the vapor phase. The concentration of all compounds released from the EO in the vapor phase throughout the incubation was measured using GC-MS analysis.

## 2. Results

### 2.1. Antibacterial Activity of EOs in Vapor Phase

Eight essential oils were preliminary evaluated in the vapor phase against 15 spoilage bacteria and 5 pathogenic bacteria. Liquid commercial EOs were deposited over the lid of the micro-well plate, away from microbial cells in the NGBA medium. In Table 1, the qualitative scores attributed to the acidification of the NGBA medium after the incubation period are reported. The non-inoculated NGBA medium exposed to different EOs did not change color, whereas the growth of all bacteria acidified the medium (yellow color). In these cases, a viability assay was further carried out to verify bacteriostatic or bactericidal activity. Bactericidal activity was marked with “0*”. All EOs, except SOO, inhibited bacterial growth at different extents (Table 1). The antibacterial activity of VOCs released from EOs differentially affected spoiler and pathogenic target strains. The viability of spoiler strains was reduced in six out of eight EOs, whereas only OVO was able to inhibit 80% of pathogens. The GO showed bactericidal activity against acetic acid bacteria. Exposure to LEO determined only partial growth inhibition against 5 out of 20 strains. TTO, OVO, and RTO were the EOs endowed with broad antibacterial activity against the tested strains. *Dickeya dadantii* LMG 25991 was the most sensitive strain to EO vapor exposure due to bactericidal activity produced by five out of eight EOs. Conversely, *Pseudomonas aeruginosa* DSM 939 was the only strain resistant to all EOs (Table 1), and for this reason, it was not additionally assayed. OVO and RTO showed bactericidal activity against eight and seven strains, respectively. OVO showed a broad antibacterial activity in the vapor phase and, in particular, bactericidal activity against four spoiler bacteria and four foodborne pathogens, as well as a bacteriostatic effect against two spoiler strains (Table 1). Although a specific sensitivity was found as in the case of LO against *Pseudomonas chicorii* ITEM 17298 or TRO and TTO against spoiler target strains, OVO produced the best antimicrobial effect, with ten bactericidal and two bacteriostatic activities out of twenty target strains.

### 2.2. Determination of MIC Values

On the basis of the results of *in vitro* antibacterial screening among different EOs, the OVO was chosen for further investigation. Minimum Inhibitory Concentration (MIC) values of OVO are reported in Table 2.

The lowest MIC value was achieved by a 5% *v*/*v* OVO solution in n-hexane against *D. dadantii* LMG 25991, whereas the highest values for 40% *v*/*v* in n-hexane were found against *Pec. carotovorum* subsp. *actinidiae* LMG 26003, *A. malorum* LMG 21419, *E. coli* ATCC 35401, and *Sal. enterica* ATCC 13311.

### 2.3. Antibacterial Activity of OVO Vapors on Food-Contact Surfaces

In order to control cross-contamination phenomena and propose a new application of essential oil vapors as decontamination agents of abiotic surfaces, the antibacterial activity of OVO vapors was evaluated on three food-contact surfaces contaminated with four foodborne pathogens. Two concentrations were used, corresponding to MIC values in the vapor phase (Table 2), against Gram-positive strains (20% *v*/*v* in n-hexane) and Gram-negative strains (40% *v*/*v* in n-hexane).

Results of viable cells of different strains laid on stainless steel, polypropylene, and glass, and exposed to air or OVO vapors for 24 h at 25 °C, are reported in Table 3.

The viable cell load of *E. coli* ATCC 35401, *Sal. enterica* ATCC 13311, and *L. monocytogenes* DSM 20600 remained stable during incubation at their initial values in the range 6.5–6.8 log cfu coupon^−1^. Conversely, a viable load of *Sta. aureus* DSM 799 significantly increased on all surfaces exposed to air.

OVO vapors at two concentrations eradicated viable cells of *E. coli* ATCC 35401, *Sal. enterica* ATCC 13311, and *L. monocytogenes* DSM 20600 laid on all food-contact surfaces, resulting in cell counts under the detection limit (200 CFU coupon^−1^). In addition, the following viability assay in broth did not produce any viable cells, lowering the lethality of the exposure to less than a single viable cell per coupon (Table 3).

Differently, *Sta. aureus* DSM 799 survived OVO exposure, with only the glass surface showing a viable cell count of 3.2 log cfu coupon^−1^ after incubation. In the case of polypropylene and stainless steel surfaces, the viability assay allowed us to estimate cell survival in the range of 1–200 cfu coupon^−1^.

The growth of *Sta. aureus* DSM 799 reached 8 log cfu coupon^−1^ on all surfaces in the presence of beef extract as a soiling agent. OVO treatment at 377 µg cm^−3^ air resulted in a complete eradication of *Sta. aureus* DSM 799 only on stainless steel coupons, while on polypropylene and glass surfaces, a reduction higher than 5 log cfu coupon^−1^ was achieved.

### 2.4. HS-GC-MS Analysis

In order to achieve qualitative and quantitative data related to the mixture of volatile compounds released by oregano essential oil producing growth inhibition, the headspace over the abiotic food-contact contaminated surfaces was analyzed during incubation.

**Figure 1:**
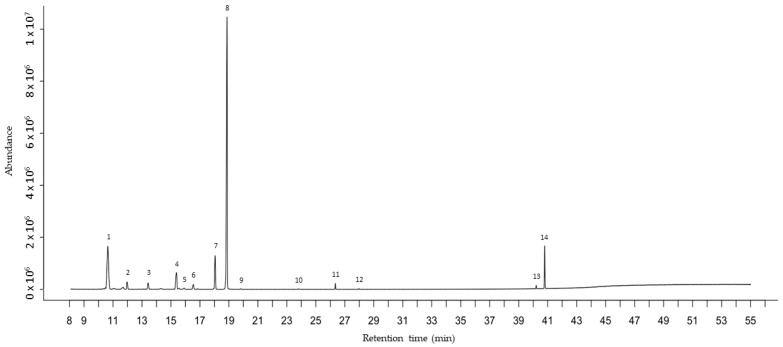
Chromatographic profile obtained by HS-GC-MS analysis of OVO vapor phase for MIC 40% at 24 h. Peak identification: 1, α-Pinene (tr = 10.6 min); 2, Camphene (tr = 12.0 min); 3, β-Pinene (tr = 13.5 min); 4, β-Myrcene (tr = 15.4 min); 5, α-Terpinene (tr = 15.9 min); 6, R-Limonene (tr = 16.5 min); 7, γ-Terpinene (tr = 18.0 min); 8, p-Cymene (tr = 18.8 min); 9, o-Cymene (tr = 19.8 min); 10, 1-Octen-3-ol (tr = 23.7 min); 11, Linalool (tr = 26.3 min); 12, Thymol methyl ether (tr = 28.0 min); 13, Thymol (tr = 40.2 min); 14, Carvacrol (tr = 40.8 min).

HS-GC-MS analysis of the OVO vapor phase employed in this paper identified 14 VOCs that, at the end of incubation, showed the concentrations reported in Table 4. An example of an HS-GC-MS chromatogram is depicted in Figure 1.

As shown in Table 4, the main VOCs were α-Pinene, p-Cymene, β-Myrcene, and Camphene, representing 90% of volatile terpenes in this essential oil. The comparison of the VOCs’ composition at 24 h showed that all the concentrations significantly increased at MIC 40% (754 µg cm^−3^ air of OVO) compared to MIC 20% (MIC at 377 µg cm^−3^ air of OVO), with the exception of Linalool, o-Cymene, and Thymol. The mean concentration of total terpenes increased from 1620 ng mL^−1^ in the MIC 20% sample to 2802 ng mL^−1^ in the MIC 40% sample (Table 4).

Changes in VOCs’ composition and their concentration during 24 h of incubation are reported in Table S1 (Supplementary Material). At 377 µg cm^−3^ air of OVO (MIC 20%), the concentration of p-Cymene, α-Pinene, Camphene, β-Pinene, β-Myrcene, α-Terpinene, Limonene, Thymol methyl ether, and 1-Octen-3-ol remained stable throughout the incubation. The concentration of γ-Terpinene and o-Cymene increased significantly at 8 h, whereas the concentration of Carvacrol, Thymol, and Linalool increased significantly at 24 h. At 754 µg cm^−3^ air of OVO (MIC 40%), only the concentration of Linalool, Thymol methyl ether, and 1-Octen-3-ol increased significantly during the incubation (Table S1).

Several VOCs at MIC40% were found at double concentration compared to those found at MIC20%, as expected. However, the concentration of Carvacrol, Linalool, and Thymol in the headspace increased during incubation but showed similar values at both MIC 20% and MIC40%.

## 3. Discussion

Essential oils are endowed with antibacterial activity both in the liquid and vapor phases [21,22]. However, methods for the large screening of antibacterial activity are available only for liquid EOs [23]. Indeed, methods developed with the aim of assessing the antibacterial activity in the vapor phase usually use different airtight apparatus or plates [7,8,9,24].

In this work, a multi-well disc volatilization method in an NGBA medium was set up to preliminary evaluate the antibacterial activity of EO vapors. Several multi-well assays were developed for the evaluation of the antimicrobial activity of volatile EO compounds in the vapor phase. Feyaerts et al. [25] developed a vapor-phase-mediated patch assay using 96-well microtiter plates to evaluate the antifungal activity of EOs in the vapor phase, but this method provided only qualitative results. Houdkova et al. [26] developed a broth microdilution volatilization method to assess the antibacterial activity of essential oil compounds in both liquid medium and vapor phases. Despite advantages such as the possibility to evaluate the *in vitro* antibacterial activity in liquid and vapor phases simultaneously, this method provides only indicative values for the concentration of the essential oil in the well’s atmosphere. Indeed, the hydrophobicity and viscosity of EOs in the broth medium affect their volatilization in the well headspace [27]. However, the broth microdilution volatilization method was used to evaluate the antibacterial activity of different *T. vulgaris* L essential oil vapors against *Sta. aureus*. MIC values ranged from 512 to 1024 µg mL^−1^ depending on the essential oil composition [27]. The application of the modified *in vitro* vapor-contact assay applied here showed that RTO and OVO, followed by TTO, were the EOs endowed with the highest antibacterial activity in the vapor phase against spoilage and pathogenic bacteria, although five spoilers and one pathogen were resistant to all EO vapors. The MIC values of OVO in the vapor phase are in accordance with the literature, as in the case of *Sta. aureus* DSM 799, which was found to be close to the range reported for a collection of *Sta. aureus* strains [28].

Liquid thyme essential oil, oregano essential oil, or Carvacrol at 0.05% *v*/*v* were able to eradicate *Salmonella typhimurium* biofilms from polystyrene and stainless steel surfaces [29]. Soap supplemented with 0.5% oregano essential oil was used to clean stainless steel, wood, and plastic surfaces contaminated with four pathogens, demonstrating the eradication of *L. monocytogenes* on wood and plastic [30]. Sengun et al. [31] treated medium-density fiberboard surfaces contaminated with *E. coli* (≅0.2 log cfu cm^−2^) with a mixture of liquid oregano, cinnamon, and clove oils (1:1:1, 10% *v*/*v*), achieving a complete reduction of the target strain and total mesophilic aerobic bacteria after 1 min of application.

Despite the well-known antimicrobial activity of EOs applied in their liquid form, limited data are available on the antimicrobial activity of essential oil vapors. The spread of microbial resistance to disinfectants and detergents has been reported for a long time in both hospital and food industry environments [32,33], highlighting the need for new environmentally friendly control strategies. For this purpose, the application of essential oils endowed with broad antiseptic properties has been largely suggested.

Food-contact surfaces can be contaminated by foodborne pathogens and spoilage bacteria. *L. monocytogenes* and *Sta. aureus* showed an average incidence ranging from 25 to 50% in the food preparation facilities of schools [34]. *L. monocytogenes*, *Salmonella* spp., and *Sta. aureus* were detected in the range of 11–26% on knives, tables, and cold rooms of sausage-processing facilities [35]. Spoilage *Pseudomonas* spp. was also found (20%) on food-contact surfaces from dairy, meat, and egg processing plants after cleaning and disinfection procedures [13]. Recently, *Pec. carotovorum* subsp. *carotovorum* showed biofilm formation on polypropylene boxes used in the vacuum cooling of vegetables [36]. As far as the persistence of foodborne pathogens on food-contact surfaces is concerned, *Sta. aureus* showed survival longer than 6 weeks on stainless steel [37] and polypropylene [38] but less than 40 days on glass [39]. *L. monocytogenes* showed a survival higher than one month on stainless steel [40]. As regards Gram-negative bacteria, *E. coli* O157:H7 persisted on oriented polypropylene for 15 days [41], whereas 14 Shiga-toxigenic *Escherichia coli* (STEC) strains were recovered from stainless steel after 30 days [42]. *E. coli* showed few days of persistence on glass surfaces, whereas *Sal. enterica* showed better resistance on stainless steel than plastic surfaces but with a strong effect of the serotype [43].

The application of essential oils in the vapor phase has been proposed to target the solid–liquid interface of all closed and open food-contact surfaces, representing the starting point of microbial attachment and colonization, as stated by Verran et al., 2008 [44].

Due to the increasing diffusion of foodborne diseases, which can be also the result of the cross-contamination of foods, we conducted further experimental activity on only pathogenic target bacteria strains.

Our results show that *Sta. aureus* DSM 799 was able to grow on stainless steel, polypropylene, and glass after 24 h of incubation at 25 °C in the presence or absence of beef extract as a soiling agent. Viable cell counts of other pathogens did not significantly increase on all food-contact materials tested. This behavior could be strain-specific, although *Sta. aureus* has been reported to grow on stainless steel and polypropylene at 25 °C with different soiling agents [45]. OVO confirmed MIC values detected in the microplate assay against *E. coli* ATCC 35401, *Sal. enterica* ATCC 13311, and *L. monocytogenes* DSM 20600 laid on the three food-contact surfaces. OVO confirmed MIC values against *Sta. aureus* DSM 799 inoculated on stainless steel with or without the soil agent and polypropylene in the absence of beef extract. On the contrary, OVO did not eradicate *Sta. aureus* DSM 799 laid on glass. Since the antibacterial action of a gaseous antimicrobial compound on food-contact surfaces is affected by the interaction with the abiotic surface and the distribution of microbial cells onto the surface [46], according to contact angle values, the hydrophilic glass [46] could negatively interact with hydrophobic monoterpenes of OVO. In addition, *Sta. aureus* DSM 799 cells could have the best fitness for glass surfaces in comparison to steel and polypropylene.

The results reported are among the few studies evaluating the chemical composition of OVO in the gaseous phase. Recently, Nakas et al. [47] found that the headspace of Greek oregano essential oil was mainly composed of Carvacrol, while γ-Terpinene, p-Cymene, and β-Myrcene were found at lower concentrations. On the contrary, our results revealed that the main components in the headspace of the plastic box used in antibacterial assays were α-Pinene, p-Cymene, β-Myrcene, and Camphene. The different compositions can be due to the different EO chemotypes, EO origin, and different conditions for the EO application in the plastic box as well as for differences in HS-GC-MS analysis. The main compound we found in the headspace was α-Pinene, a hydrocarbon monoterpene endowed with antibacterial activity. *Baccaris reticulata* EO, with 31% α-Pinene, showed an MIC, determined by microdilution method, of 1000 µg mL^−1^ against *Sta. aureus* [48]. Wang et al. [49] reported MIC values of α-Pinene of 0.4–0.6 mg mL^−1^ against *E. coli*, *Sal. enterica* and *Sta. aureus* in wine. As regards the mechanism of action, α-Pinene alters the permeability of cell membranes, interferes with metabolic pathways, and is a modulator of antibiotic resistance [50]. Other monoterpenes found in OVO, such as Carvacrol, Thymol, p-Cymene, and γ-Terpinene, cause a perturbation of the lipidic fraction of the bacterial membranes and can penetrate the cell membrane, interacting with intracellular components [51].

The concentration of VOCs in the headspace of food-contact surfaces contaminated with pathogens was measured by HS-GC-MS analysis during a 24 h incubation. Generally, for most of the compounds, their highest concentration was recorded at 8 h, suggesting that they were completely released in the first hours of the incubation. In the case of Carvacrol, Thymol, and Linalool a slight increase in their concentration was observed throughout incubation. In addition, some compounds did not double their concentration when the experiment was carried out at 754 µg cm^−3^ air instead of 377 µg cm^−3^ air.

These data underline that the liquid–vapor equilibrium of volatile compounds released from a liquid mixture, as is normal for EO, undergoes several parameters, including the solvent defined for different experiments, as demonstrated for the mixtures citronellal/geraniol and citronellal/citronellol at different vacuum pressures [52] or eugenol/caryophyllene at reduced pressures [53].

It is arguable that the antimicrobial activity of EOs in the vapor phase is affected by changes in the liquid–vapor equilibria occurring for each of the VOCs composing EOs.

## 4. Materials and Methods

### 4.1. Chemical Reagents and Essential Oils

Chemical standards (Thymol, Carvacrol, p-Cymene and γ-Terpinene) and a 2.5 mL SGE™ gas-tight syringe equipped with a push-pull valve with a Luer Lock (SGE Analytical Science, Ringwood, Australia) were purchased from Sigma-Aldrich (St. Louis, MO, USA). A mixture of chemical standards (α-Pinene, Camphene, β-Pinene, β-Myrcene, α-Terpinene, R-Limonene, o-Cymene, 1-Octen-3-ol and Linalool) were purchased from Ultra Scientific Italia S.r.l. (Bologna, Italy). A mixture of normal alkanes (C5–C29) was purchased from o2si smart solutions (Charleston, SC, USA). Hexane and methanol were purchased from Carlo Erba Reagents (Milan, Italy). Helium at a purity of 99.9995% was obtained from Sapio S.r.l. (Bari, Italy). Commercially available red thyme essential oil (RTO, *Thymus vulgaris* L.) and oregano essential oil (OVO, *Origanum vulgare* L.) were purchased from Bristol Botanicals Ltd. (Bristol, UK), and lavender essential oil (LO, *Lavandula angustifolia* Mill.) and tea tree essential oil (TTO, *Melaleuca alternifolia* Maiden & Betche C.) were purchased from Sigma-Aldrich S.r.l. (Milan, Italy). Rosemary essential oil (RO, *Rosmarinus officinalis* L.), sweet orange essential oil (SOO, *Citrus sinensis* L. Osbeck), grapefruit essential oil (GO, *Citrus grandis* L. Osbeck) and lemon essential oil (LEO, *Citrus limon* L. Osbeck) were purchased from Farmalabor S.r.l. (Canosa di Puglia, Apulia, Italy). Essential oils (EOs) were stored at 4 °C in dark bottles prior to their use. According to the information given by the supplier, RTO, OVO, LO, TTO, and RO were produced by a steam distillation method; SOO and GO were produced by a cold-pressed peel method; and LEO was produced bya a cold-pressed fruit method.

### 4.2. Bacterial Strains and Growth Conditions

The antibacterial activity of EO vapors was assessed against 15 spoiler and 5 pathogenic bacteria. Bacteria were previously isolated from plant foods or were purchased from international bacterial collections, including the BCCM/LMG Bacteria Collection, Leibniz-Institut DSMZ, the German Collection of Microorganisms and Cell Cultures, and the Agro-Food Microbial Culture Collection (ITEM) at the Institute of Sciences of Food Production of Bari, Italy (http://server.ispa.cnr.it/ITEM/Collection/, accessed on 4 March 2024).

Spoilage bacteria included *Erwinia persicina* ITEM 17997, *Enterobacter aerogenes* ITEM 17998, *Serratia marcescens* ITEM 17999 [54], *Pseudomonas fluorescens* NCPPB 1964^T^, *P. fluorescens* L1A [55], *P. putida* ITEM 17297, *P. chicorii* ITEM 17298 [56], *Acetobacter syzygii* LMG 21419 [57], *Gluconobacter oxydans* LMG 1408, *Gluconacetobacter saccharivorans* LMG 1582 [58], *P. marginalis* pv. *marginalis* LMG 2210, *Pectobacterium carotovorum* subsp. *actinidiae* LMG 26003, *P. carotovorum* subsp. *carotovorum* LMG 2404, *Pantoea agglomerans* LMG 2565, and *Dickeya dadantii* LMG 25991. *Enterobacteriaceae* and *Pseudomonadaceae* were grown in mPlate Count Broth (mPCB, Becton Dickinson Italia, Milan, Italy) for 24 h at 30 °C, whereas acetic acid bacteria were grown in YPM broth (D-mannitol 2.5%; yeast-extract 0.5%; peptone 0.3%) for 72 h at 30 °C.

Pathogenic bacteria included *Staphylococcus aureus* DSM 799, *Escherichia coli* ATCC 35401, *Pseudomonas aeruginosa* DSM 939, *Listeria monocytogenes* DSM 20600, and *Salmonella enterica* ATCC 13311. Bacterial strains were grown in Brain Heart Infusion broth (BHI, Biolife Italiana Srl, Milan, Italy) for 24 h at 37 °C.

### 4.3. Vapor Contact Assay

**Figure 2:**
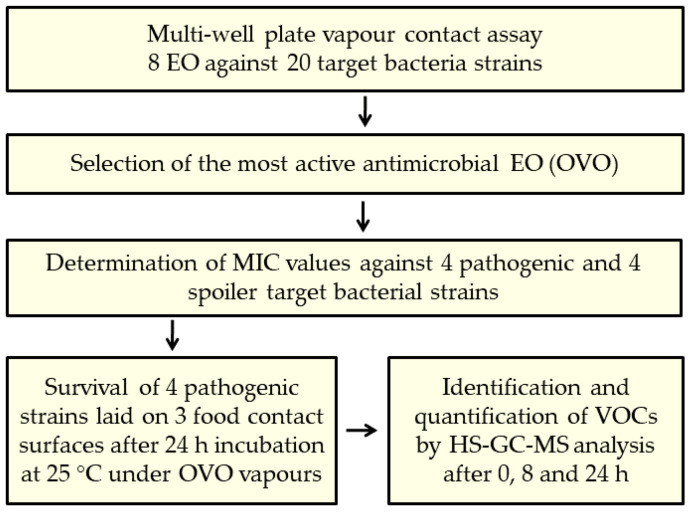
Experimental set up followed to define the most promising EO vapors to control microbial contamination of food-contact surfaces.

The experimental activity carried out in this work is depicted in Figure 2.

In this work, a multi-well plate vapor-contact assay was developed to evaluate the antibacterial activity of essential oils. In particular, 12-well polystyrene plates (BD Falcon™, Becton Dickinson International, Erembodegem, Belgium) were filled (2 mL well^−1^) with the differential medium, NGBA, used by Seo et al. [9], which changes color in response to a decrease in pH caused by the growth of glucose-fermenting microorganisms. This medium is composed of nutrient agar, 1% (*w*/*v*) d-glucose anhydrous, and 0.025% (*w*/*v*) bromocresol purple as a pH indicator. The NGBA medium was sterilized, kept at 50 °C, and placed in each well (2 mL). Ten microliters of each bacteria suspension (ca. 7.0 log cfu mL^−1^) were deposited on the solidified NGBA and kept in a laminar flow biosafety hood at 25 °C for 30 min. A sterile paper disc (diameter 8 mm, Biolife Italiana S.r.l., Milan, Italy) was attached to each of the wells of the plate cover. Then, 8 μL of liquid EOs was deposited in each well. An un-inoculated well served as the negative control, whereas an inoculated well exposed to n-hexane served as the positive control. In order to avoid the transfer of EO vapors among wells of the same micro-well plate, a heat-reflective mat in polyethylene resin (Geko Reflex, Tre Emme Spa, Sant’Angelo in Pontano, Italy) was shaped with a hole-punch cutter tool and attached to the plate cover. Immediately after depositing diluted liquid EO on the paper disc, the plate was closed with the cover, sealed tightly using Parafilm, and incubated at 30 °C or 37 °C for 48 h. After incubation, microbial growth was determined by assessing the color change in NGBA. A color change from purple to yellow indicated bacterial growth (Figure S1).

The most active EO, OVO, among the eight here assayed, was selected for the determination of the Minimum Inhibitory Concentration (MIC) in the vapor phase. Paper discs in the plate wells were soaked with 8 µL of, OVO diluted or not in n-hexane (100%, 80%, 40%, 20%, 10%, 5% *v*/*v*). The MIC was determined against *P. putida* ITEM 17297, *Pec. carotovorum* subsp. *carotovorum* LMG 2404, *D. dadantii* LMG 25991, and *A. malorum* LMG 21419 representative of spoilage bacteria, and *E. coli* ATCC 35401, *L. monocytogenes* DSM 20600, *Sta. aureus* DSM 799, and *Sal. enterica* ATCC 13311, representative of pathogenic bacteria. The MIC was defined as the lowest concentration completely inhibiting the growth of the tested bacteria (purple medium).

### 4.4. Application of EOs on Food-Contact Surfaces

The antibacterial activity of oregano essential oil vapors was assessed against four foodborne pathogens laid on three food-contact surfaces. Stainless steel, glass, and polypropylene were provided by the enterprise described in de Candia et al. [14], complying with the Framework Regulation (EC) 1935 (Commission Regulation (EC), 2004) on materials and articles intended to come into contact with food. Stainless steel, polypropylene, and glass coupons (4.84 cm^2^) were sterilized by autoclaving (121 °C for 15 min). *E. coli* ATCC 35401, *L. monocytogenes* DSM 20600, *Sal. enterica* ATCC 13311, and *Sta. aureus* DSM 799 were inoculated in 10 mL of Brain Heart Infusion broth for 24 h at 37 °C. Then, cells adjusted at an OD_600_ value of 0.3 ± 0.05 (ca. 8 log CFU mL^−1^) were centrifuged (5000× *g* for 5 min) and washed in sterile saline solution (9 g L^−1^ NaCl). A volume of fifty microliters of this suspension was evenly distributed over coupons with a sterile loop, excluding 2 mm of the edge. Coupons were allowed to dry in a sterile biohazard cabinet for ca. 30 min and then placed in Petri dishes at the bottom of a high-density polyethylene box with an internal volume of 600 mL (Ref. 11673, Albero Forte Composite s.l., Banyeres de Mariola, Spain). Oregano essential oil was diluted in n-hexane at 40% for *E. coli* and *Sal. enterica*, and at 20% for *L. monocytogenes* and *Sta. aureus,* which resulted in the MICs after previous assays. A volume of 1.2 mL was distributed on filter paper attached to the lid of the plastic box. The volume of OVO dilution in n-hexane laid in the paper disk was calculated to reach, in plastic boxes, the same concentration used for the micro-well plate assays.

Then, the boxes were sealed with Parafilm and incubated at 25 °C for 24 h. Coupons exposed to air were used as controls. After incubation, viable cells from each surface were recovered in 20 mL of sterile saline solution by stirring the coupons using an orbital shaker for 1 min at 200 rpm. Cell suspensions were decimally diluted in sterile saline solution and plated (0.1 mL) onto Tryptone bile X-glucuronide agar (TBX), Agar Listeria Ottaviani and Agosti (ALOA), Xylose-Lysine-Desoxycholate agar (XLD), and Baird–Parker agar (BP) for *E. coli* ATCC 35401, *L. monocytogenes* DSM 20600, *Sal. enterica* ATCC 13311, and *Sta. aureus* DSM 799, respectively (detection limit of 200 CFU coupon^−1^). All media were purchased from Biolife Italiana S.r.l., Milan, Italy. In order to lower the detection limit, an enrichment step was carried out: 20 mL of the saline solution used to recover bacteria from the coupons was inoculated into 20 mL of Brain Heart Infusion medium (2×). Flasks were incubated at 37 °C for 24 h. Then, a loopful of broth culture was plated on a selective medium and incubated at 37 °C for 24 h. The detection limit of this procedure was estimated as 1 CFU coupon^−1^. Populations were indicated as not detected (ND) in the absence of colonies after enrichment and plating on selective media.

**Figure 3:**
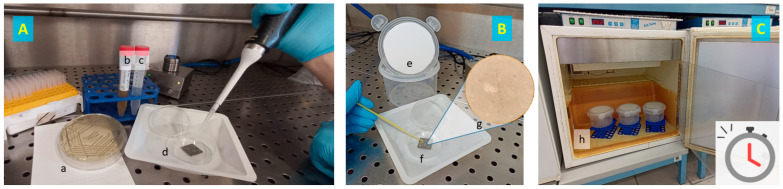
Main steps of evaluation of the antibacterial activity of OVO vapors on food-contact surfaces. (**A**) *St. aureus* DSM 799 (a) after growth in BHI broth (b) is diluted in sterile saline solution to OD_600nm_ = 0.3 (c) and then inoculated over a stainless steel surface (d). (**B**) the lid of the plastic box is covered by filter paper (e) in order to be loaded with OVO solution far from food-contact surfaces contaminated with the microbial target (f), *St. aureus* DSM 7799 in the picture (g). (**C**) Three replicates for each sample (h), and for each food-contact surface, once loaded with OVO solution, are sealed and incubated for 24 h at 25 °C when the vapor phase is sampled at 0, 8, and 24 h of incubation.

Figure 3 shows some steps of the evaluation of the contamination of food-contact surfaces followed by OVO treatment.

### 4.5. Headspace-Gas-Chromatography-Mass-Spectrometry (HS-GC-MS) Analysis

Volatile organic compounds (VOCs) were identified and quantified by HS-GC-MS analysis, as previously described by Pinto et al. [2], with some modifications. In particular, a total of 18 HS-GC-MS analyses were performed at 0 (30 min after plastic boxes sealing), 8, and 24 h of exposure at 25 °C (in triplicate) for each EO concentration (20 and 40% in hexane, see Section 4.4) using a gas-tight syringe and a GC-MS system composed of a GC (Agilent 7890A, Agilent Technologies, Palo Alto, CA, USA) coupled to a mass spectrometer (Agilent 5975C inert MSD, Agilent Technologies) and hyphenated with a Triple-Axis HED-EM detector. A volume of 1 mL of headspace was collected from the center of the HDPE plastic box (see Section 4.4) with a PTFE/GC–MS Grade Ultra Pure Silicone Septa attached to the outer surface of the plastic box at a 1 cm height from the base. The headspace sample was then injected into the GC unit using a split/split-less inlet liner (Agilent Technologies, Palo Alto, CA, USA) at a split ratio of 10. The GC unit was equipped with a VF-WAXms fused-silica capillary column (60 m × 0.25 mm i.d., 0.25 µm film thickness, Agilent Technologies). The temperature program started at 40 °C, raised to 240 °C after 5 min at a rate of 5 °C min^−1^, and was finally held at 240 °C for 10 min. The total chromatographic run time was 55 min. The temperature of the inlet, ion source and quadrupole was 250, 290 and 150 °C, respectively, and the gas carrier (helium) flow was set at 1 mL min^−1^. Electron impact Ionization (EI+) mode with an electron energy of 70 eV was used, and the mass spectra were recorded in the *m*/*z* range of 40–300 Th. The identification of compounds was carried out by comparing experimental mass spectral data with those reported in the NIST/EPA/NIH Mass Spectral Database library (National Institute of Standards and Technology, Version 2.0f, 2008, Gaithersburg, MD, USA), using a match quality higher than 80 and comparing the retention time and the mass spectral of a standard solution. The identity of volatile compounds was also confirmed by comparing their linear retention indices (LRIs), determined in relation to the retention times of C5–C29 n-alkane series, with those reported in the literature [59]. The total ion peak area of the compounds was determined by using MSD Chemstation (Agilent Technologies). The concentration of each compound was calculated by means of area interpolation on the calibration curve, which was built using a standard solution prepared in hexane or methanol. An aliquot (1 µL) of standard solutions was analyzed at a split ratio of 100. The selective ion monitoring mode was exploited using the *m*/*z* quantifier ions 93, 93, 93, 69, 121, 68, 93, 119, 119, 57, 71, 149, 135 and 135 for α-Pinene, Camphene, β-Pinene, β-Myrcene, α-Terpinene, R-Limonene, γ-Terpinene, p-Cymene, o-Cymene, 1-Octen-3-ol, Linalool, Thymol methyl ether, Thymol and Carvacrol, respectively. The *m*/*z* qualifier ions used were: 41 and 67 for β-Myrcene; 69 and 121 for β-Pinene; 72 and 85 for 1-Octen-3-ol; 77 and 121 for α-Pinene; 79 and 121 for Camphene; 91 and 134 for p-Cymene and o-Cymene; 91 and 164 for Thymol methyl ether; 91 and 150 for Thymol and Carvacrol; 93 and 107 for R-Limonene; 93 and 121 for Linalool; 93 and 136 for α-Terpinene; and 121 and 136 for γ-Terpinene.

### 4.6. Statistical Analysis

The effect of incubation time and EO treatment on the bacterial loads was evaluated through SPSS software (SPSS, Inc., Chicago, IL, USA). Tukey’s test was used to differentiate mean values at the 95% confidence interval. One-way ANOVA analysis was applied to evaluate the effect of the time of incubation on the concentration of volatile organic compounds (*p* ≤ 0.05). Least Significant Difference (LSD) values were calculated to differentiate mean values. Tukey’s test was used to differentiate mean values of the concentration of volatile organic compounds between the samples, at a 95% confidence interval.

## 5. Conclusions

In conclusion, in this work, the antibacterial activity of different essential oils in the vapor phase was evaluated through a multi-well assay. Oregano EO showed antibacterial activity against Gram-positive and Gram-negative bacteria. At the MIC, oregano EO reduced the viable cells of different bacterial pathogens on stainless steel, polypropylene, and glass surfaces. The main EO volatile compounds found in the headspace of the boxes were α-Pinene and p-Cymene. This study contributes to the development of novel strategies for the decontamination of food-contact surfaces, though the efficacy of EO vapors needs to be carefully evaluated for some main parameters, such as microbial targets and the type of contact surface. In addition, it should be considered that antimicrobial VOCs active after *in vitro* trials could be partially active under real conditions, as in the case of food applications, where the natural microbial population includes both sensitive and resistant strains. Further research is necessary to shed light on the antibacterial activity of single terpenes found in oregano EO, as well as the occurrence of interactive effects between major and minor volatile compounds.

## Figures and Tables

**Table 1:** Qualitative scores attributed to the growth of bacteria on NGBA after the exposure to essential oils vapors (2 µL mL air^−1^) for 48 h at 30 °C or 37 °C. Description of values: 5, complete acidification; 3, partial acidification; 0, no acidification.

	Target Strains	GO	LEO	LO	OVO	RO	RTO	SOO	TTO
*Spoilers*	*Acetobacter malorum* LMG 1746	0*	5	5	0*	5	0*	5	0
	*A. syzygii* LMG 21419	0*	5	5	5	5	0*	5	0*
	*Dickeya dadantii* LMG 25991	5	3	0*	0*	0*	0*	5	0*
	*Enterobacter aerogenes* ITEM 17998	5	5	5	5	5	3	5	5
	*Erwinia persicina* ITEM 17997	5	5	5	5	5	3	5	5
	*Gluconobacter oxydans* LMG 1408	0*	5	5	5	5	0*	5	0
	*Pantoea agglomerans* LMG 2565	5	5	5	5	5	5	5	5
	*Pectobacterium carotovorum* subsp. *actinidiae* LMG 26003	5	3	5	0*	0	0*	5	0*
	*Pec. carotovorum* subsp. *carotovorum* LMG 2404	5	3	5	0*	0	0*	5	0*
	*Pseudomonas chicorii* ITEM 17298	5	5	0*	3	5	3	5	3
	*Pse. fluorescens* ITEM 19245	5	5	5	0	0	0	5	0
	*Pse. fluorescens* NCPPB 1964^T^	5	5	5	3	5	3	5	0
	*Pse. marginalis* pv. *marginalis* LMG 2210	5	5	5	5	5	3	5	3
	*Pse. putida* ITEM 17297	5	3	5	0	5	0	5	5
	*Serratia marcescens* ITEM 17999	5	3	5	5	5	5	5	3
*Pathogens*	*Escherichia coli* ATCC 35401	5	5	5	0*	5	5	5	5
	*Listeria monocytogenes* DSM 20600	5	5	5	0*	5	5	5	5
	*Pse. aeruginosa* DSM 939	5	5	5	5	5	5	5	5
	*Salmonella enterica* ATCC 13311	5	5	5	0*	5	5	5	3
	*Staphylococcus aureus* DSM 799	5	5	5	0*	5	0*	5	5

0* bactericidal activity detected after incubation for 48 h at 30 °C on Plate Count Agar.

**Table 2:** Minimum Inhibitory Concentration (MIC) of OVO at different concentrations in n-hexane and estimated MIC in air, against selected spoilage and pathogenic bacteria after 24 h of incubation at 30 °C or 37 °C.

Target Strain	MIC of OVO	
	as *v*/*v* in n-Hexane *v*/*v*	in µg cm^−3^ Air
*A. malorum* LMG 21419	40%	753.6
*D. dadantii* LMG 25991	5%	94.2
*E. coli* ATCC 35401	40%	753.6
*L. monocytogenes* DSM 20600	20%	376.8
*Pec. carotovorum* subsp. *actinidiae* LMG 26003	40%	753.6
*Pec. carotovorum* subsp. *carotovorum* LMG 2404	10%	188.4
*Sal. enterica* ATCC 13311	40%	753.6
*Sta. aureus* DSM 799	20%	376.8

**Table 3:** Viable cells (log cfu coupon^−1^) of pathogens on food-contact surfaces after 24 h at 25 °C in air or under oregano essential oil (OVO) vapors at their MIC values.

Strain	Initial Load	Treatment	Stainless Steel	Polypropylene	Glass
*Escherichia coli* ATCC 35401	6.55 ± 0.02 ^A^	Air	6.98 ± 0.20 ^Aa^	6.97 ± 0.12 ^Aa^	6.76 ± 0.12 ^Aa^
		OVO ^1^	n.d. ^Bb^	n.d. ^Bb^	n.d. ^Bb^
*Listeria monocytogenes* DSM 20600	6.80 ± 0.20 ^A^	Air	6.79 ± 0.17 ^Aa^	6.72 ± 0.10 ^Aa^	6.74 ± 0.12 ^Aa^
		OVO ^2^	n.d. ^Bb^	n.d. ^Bb^	n.d. ^Bb^
*Salmonella enterica* ATCC 13311	6.72 ± 0.10 ^A^	Air	6.94 ± 0.02 ^Aa^	7.12 ± 0.06 ^Aa^	6.44 ± 0.19 ^Aa^
		OVO ^1^	n.d. ^Bb^	n.d. ^Bb^	n.d. ^Bb^
*Staphylococcus aureus* DSM 799	6.74 ± 0.09 ^A^	Air	7.81 ± 0.14 ^Ba^	7.66 ± 0.02 ^Ba^	7.45 ± 0.07 ^Ba^
		OVO ^2^	n.d. ^Bb^	n.d. ^Bb^	3.23 ± 0.02 ^Bb^

n.d.: not detected (detection limit of 200 CFU coupon^−1^). ^1^, MIC at 754 µg cm^−3^ air of OVO; ^2^, MIC at 377 µg cm^−3^ air of OVO. Tukey test (*p* ≤ 0.05) was applied to differentiate mean values. Different uppercase letters indicate significant differences, for each strain, within rows, whereas different lowercase letters indicate significant differences, for each strain, within each food-contact material.

**Table 4:** HS-GC-MS analysis of OVO vapor phase at the MIC levels after 24 h at 25 °C.

Compounds	LRIlt/LRIsp ^a^	LOD ^b^ (ng mL^−1^)	377 µg cm^−3^ Air		754 µg cm^−3^ Air	
			Mean ^c^ Concentration (ng mL^−1^)	Mean ^c^ Composition (%)	Mean ^c^ Concentration (ng mL^−1^)	Mean ^c^ Composition (%)
α-Pinene ^d^	1020/1021	9	1009a ± 96	62	1763b ± 69	63
p-Cymene ^d^	1270/1275	0.17	343a ± 17	21	582b ± 59	21
β-Myrcene ^d^	1160/1167	0.76	63a ± 4	3.9	132b ± 18	4.7
Camphene ^d^	1062/1067	1.5	62a ± 8	3.8	113b ± 7	4.0
β-Pinene ^d^	1120/1109	1.6	29a ± 5	1.8	61b ± 5	2.2
γ-Terpinene ^d^	1250/1251	0.10	29a ± 2	1.8	56b ± 8	2.0
Carvacrol ^d^	2225/2225	0.24	46a ± 1	2.9	48a ± 6	1.7
R-Limonene ^d^	1200/1203	0.50	12a ± 1	0.72	19a ± 2	0.69
Linalool ^d^	1551/1551	0.40	16a ± 2	1.0	14a ± 1	0.51
α-Terpinene ^d^	1180/1183	1.3	4.7a ± 0.1	0.29	7.5b ± 0.7	0.27
o-Cymene ^d^	1298/1309	0.73	2.7a ± 0.1	0.17	3.1a ± 0.1	0.11
Thymol ^d^	2198/2193	0.19	2.8a ± 0.1	0.17	2.9a ± 0.4	0.10
Thymol methyl ether ^e^	1599/1616	0.12	0.5a ± 0.1	traces ^f^	0.7a ± 0.1	traces ^f^
1-Octen-3-ol ^d^	1450/1450	0.13	0.38a ± 0.05	traces ^f^	0.34a ± 0.04	traces ^f^
Total terpenes			1620		2802	

^a^ LRIIt: Linear retention index reported in the literature at www.nist.gov (accessed on 4 March 2024); LRIsp: Linear retention index calculated using n-alkanes (C5–C29) with a VF-WAXms column; ^b^ LOD: limit of detection; ^c^ n = 3; ^d^ Volatile organic compounds identified with chemical standards; ^e^ Thymol methyl ether was quantified by comparing with the calibration curve of Thymol; ^f^ Compound percentage < 0.1%. Tukey test was applied to differentiate mean values between the two concentrations of OVO at 24 h of incubation. Different lowercase letters indicate differences between mean values within rows (*p* ≤ 0.05).

## Data Availability

The Data are contained in the manuscript and Supplementary Materials.

## Supplementary Materials

The following are available online at https://www.mdpi.com/article/10.3390/antibiotics13040371/s1, Table S1: Mean concentration (ng mL^−1^) of volatile compounds in boxes with 377 µg cm^−3^ air or 754 µg cm^−3^ air of OVO, during 24 h at 25 °C. Figure S1: Color of NGBA medium inoculated with bacterial strains and exposed to essential oil vapors for 24 h at 30 °C or 37 °C: (a) bacterial growth, complete acidification; (b) partial bacterial growth, partial acidification; (c) no bacterial growth, no acidification, which were scored 5, 3 and 0, respectively.
