# Supplementary material for: Antibacterial Activity of Oregano (Origanum vulgare L.) Essential Oil Vapors against Microbial Contaminants of Food-Contact Surfaces
Source: Antibiotics (Basel). 2024 Apr 18;13(4):371. doi: 10.3390/antibiotics13040371 (PMC11047463; doi:10.3390/antibiotics13040371)
Supplement: Supplementary file 1 [file antibiotics-13-00371-s001.zip › antibiotics-2924430-SI.pdf]

antibiotics

Article

Antibacterial Activity of Oregano (*Origanum vulgare* L.) Essential Oil Vapors against Microbial Contaminants of Food-Contact Surfaces

Loris Pinto, Salvatore Cervellieri, Thomas Netti, Vincenzo Lippolis and Federico Baruzzi *

Institute of Sciences of Food Production, National Research Council of Italy, Via G. Amendola 122/O, 70126 Bari, Italy; th.netti97@gmail.com (T.N.); salvatore.cervellieri@ispa.cnr.it (S.C.); vincenzo.lippolis@ispa.cnr.it (V.L.); federico.baruzzi@ispa.cnr.it (F.B.)
* Correspondence: federico.baruzzi@ispa.cnr.it (F.B.); Tel.: +39-080-5929319

Supplementary Materials

Table S1. Mean concentration (ng mL^{-1}) of volatile compounds in boxes with 377 µg cm^{-3} air or 754 µg cm^{-3} air of OVO, during 24 h at 25 °C.

		**Concentration (ng mL^{-1})**													
**Concentration of OVO**	**Time**	**γ-Terpinene**	**p-Cymene**	**Carvacrol**	**Thymol**	**α-Pinene**	**Camphene**	**β-Pinene**	**β-Myrcene**	**α-Terpinene**	**Limonene**	**o-Cymene**	**Linalol**	**Thymol methyl ether**	**1-octen–3-ol**
377 µg cm^{-3} air	0 h	29,22a ± 1,48	379,22a ± 40,57	15,85a ± 2,28	1,26a ± 0,09	1564,98a ± 467,40	93,64a ± 30,57	43,69a ± 15,20	84,25a ± 16,10	5,53a ± 0,44	13,28a ± 0,99	2,65a ± 0,06	5,37a ± 1,71	0,23a ± 0,09	0,11a ± 0,07
	8 h	46,44b ± 5,64	506,98a ± 56,59	34,58b ± 1,31	2,31b ± 0,18	1401,91a ± 31,86	88,53a ± 2,25	45,34a ± 1,61	106,16a ± 14,44	6,37a ± 0,54	16,56a ± 1,49	2,92b ± 0,08	12,53b ± 1,80	0,52a ± 0,12	0,31a ± 0,06
	24 h	29,01a ± 1,99	343,23a ± 16,72	46,17c ± 1,23	2,80b ± 0,12	1008,92a ± 95,81	62,06a ± 8,27	28,61a ± 5,32	63,45a ± 4,28	4,72a ± 0,14	11,59a ± 0,65	2,67a ± 0,02	14,30b ± 0,96	0,65a ± 0,09	0,34a ± 0,04
754 µg cm^{-3} air	0 h	52,55a ± 23,15	555,84a ± 198,02	18,91a ± 7,99	1,43a ± 0,51	2234,92a ± 381,50	121,75a ± 33,23	67,86a ± 22,63	138,57a ± 56,79	7,64a ± 2,15	19,71a ± 6,87	2,92a ± 0,31	4,49a ± 0,32	0,16a ± 0,03	0,06a ± 0,01
	8 h	71,98a ± 14,38	700,68a ± 113,21	34,98a ± 8,24	2,29a ± 0,42	2295,94a ± 351,81	137,72a ± 24,35	78,35a ± 15,35	172,65a ± 35,65	9,03a ± 1,31	24,10a ± 4,19	3,27a ± 0,19	14,33b ± 0,10	0,51b ± 0,02	0,36b ± 0,01
	24 h	55,85a ± 8,33	582,12a ± 59,36	48,34a ± 6,18	2,91a ± 0,39	1763,33a ± 69,18	112,77a ± 6,53	61,50a ± 4,92	132,33a ± 17,51	7,46a ± 0,72	19,32a ± 2,24	3,07a ± 0,11	15,63b ± 1,63	0,48b ± 0,06	0,38b ± 0,05

One-way ANOVA analysis ($P \leq 0.05$) was applied to differentiate mean values. The least significant difference (LSD) values were calculated to separate mean values for each compound, at each concentration of OVO: 377 µg cm^{-3} air of OVO, γ-Terpinene, 13,97 ng mL^{-1}; p-Cymene 164,95 ng mL^{-1}; carvacrol 6,39 ng mL^{-1}; thymol 0,59 ng mL^{-1}; α-Pinene 1102,93 ng mL^{-1}; camphene 73,31 ng mL^{-1}; β-Pinene 36,59 ng mL^{-1}; β-Myrcene 51,03 ng mL^{-1}; α-Terpinene 1,62 ng mL^{-1}; limonene 3,76 ng mL^{-1};o-Cymene 0,23 ng mL^{-1}; linalol 6,13 ng mL^{-1}; thymol methyl ether 0,44 ng mL^{-1}; 1-octen-3-ol 0,29 ng mL^{-1}; 754 µg cm^{-3} air of OVO, γ-Terpinene, 66,25 ng mL^{-1}; p-Cymene 543,28 ng mL^{-1}; carvacrol 31,07 ng mL^{-1}; thymol 1,68 ng mL^{-1}; α-Pinene 1206,82 ng mL^{-1}; camphene 95,95 ng mL^{-1}; β-Pinene 63,16 ng mL^{-1}; β-Myrcene 160,41 ng mL^{-1}; α-Terpinene 6,08 ng mL^{-1}; limonene 18,88 ng mL^{-1};o-Cymene 0,85 ng mL^{-1}; linalol 3,78 ng mL^{-1}; thymol methyl ether 0,19 ng mL^{-1}; 1-octen-3-ol 0,13 ng mL^{-1}. Different lowercase letters indicate significant differences within columns at each concentration of OVO.

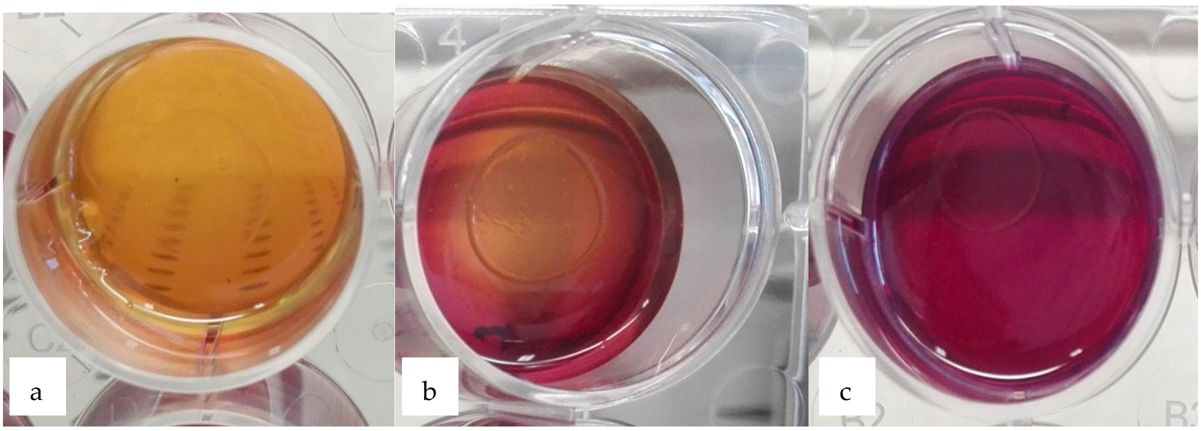

Figure S1. Colour of NGBA medium inoculated with bacterial strains and exposed to essential oil vapours for 24 h at 30°C or 37 °C: (**a**) bacterial growth, complete acidification; (**b**) partial bacterial growth, partial acidification; (**c**) no bacterial growth, no acidification that were scored 5, 3 and 0, respectively.
